# Expression and activity profiles of DPP IV/CD26 and NEP/CD10 glycoproteins in the human renal cancer are tumor-type dependent

**DOI:** 10.1186/1471-2407-10-193

**Published:** 2010-05-11

**Authors:** Adolfo Varona, Lorena Blanco, Itxaro Perez, Javier Gil, Jon Irazusta, José I López, M Luz Candenas, Francisco M Pinto, Gorka Larrinaga

**Affiliations:** 1 Department of Physiology, Faculty of Medicine and Dentistry, University of the Basque Country, Barrio Sarriena s/n, 48940-Leioa, Spain; 2 Department of Anatomic Pathology, University Hospital of Cruces, Plaza de Cruces s/n, 48903-Cruces/Barakaldo, Spain; 3 Institute for Chemical Research, CSIC-Isla de la Cartuja, Avd Americo Vespucio 49, 41092-Sevilla, Spain

## Abstract

**Background:**

Cell-surface glycoproteins play critical roles in cell-to-cell recognition, signal transduction and regulation, thus being crucial in cell proliferation and cancer etiogenesis and development. DPP IV and NEP are ubiquitous glycopeptidases closely linked to tumor pathogenesis and development, and they are used as markers in some cancers. In the present study, the activity and protein and mRNA expression of these glycoproteins were analysed in a subset of clear-cell (CCRCC) and chromophobe (ChRCC) renal cell carcinomas, and in renal oncocytomas (RO).

**Methods:**

Peptidase activities were measured by conventional enzymatic assays with fluorogen-derived substrates. Gene expression was quantitatively determined by qRT-PCR and membrane-bound protein expression and distribution analysis was performed by specific immunostaining.

**Results:**

The activity of both glycoproteins was sharply decreased in the three histological types of renal tumors. Protein and mRNA expression was strongly downregulated in tumors from distal nephron (ChRCC and RO). Moreover, soluble DPP IV activity positively correlated with the aggressiveness of CCRCCs (higher activities in high grade tumors).

**Conclusions:**

These results support the pivotal role for DPP IV and NEP in the malignant transformation pathways and point to these peptidases as potential diagnostic markers.

## Background

Although never demonstrated in human, a wide variety of factors have been reported to be involved in renal cancer development in experimental animals [1]. Clinical data support that Renal Cell Carcinomas (RCCs) are neoplasms with high prevalence and mortality rates [2]. Histologically, they represent a heterogeneous group of tumors with different behavior and prognosis ranging from benign tumors to extremely aggressive cancers who have been reclassified in the last WHO classification of renal tumors [3]. However, the underlying phenomena related with the wide prognostic spectrum of this group of tumors are a permanent matter of debate far to be understood. To date, there is no clinical marker to detect the disease in the asymptomatic potentially curable phase nor to predict with reliability the clinical course of every case. Only classical parameters like histological type, stage and grade may help for such a purpose, but depending on the clinical setting and other patient's circumstances, many individual cases often escape the general rules of tumor behavior making necessary the discovering of more predictable parameters.

The increased knowledge of these tumors has led to the implication of several proteinases in its genesis, growth and dissemination, and most efforts have been directed towards the understanding of the role of matrix metalloproteinases [4,5]. However, very little is known about the implication of other proteinases such as peptidases.

Several peptidases are well-known membrane-bound glycoproteins which present a demonstrated potential as prognostic and diagnostic markers in solid tumors. Among them, two glycopeptidases have been broadly related to exert pivotal roles in cancer pathophysiology; dipeptidyl peptidase IV (DPP IV), identical with CD26 or gp110-EC 3.4.14.5-, and neutral endopeptidase (NEP), also CD10 or CALLA glycoprotein-EC 3.4.24.11 - [6-8].

Normally, DPP IV and NEP act as regulatory proteins in cancer progression and development by modulating the effects of biologically active peptides, but eventually, they also can act as proteinases which execute extracellular matrix degradation [6,7].

DPP IV is a 110-kDa ectoenzyme that belongs to the serine protease family. It is widely expressed in endothelial and epithelial cells, several critical chemokines and cytokines being its natural substrates [9].

NEP is a 90-110 kDa membrane-bound glycoprotein which is normally expressed in most mammalian tissues and belongs to the M13 family of zinc peptidases. Natural substrates for NEP are enkephalins, angiotensins, bradykinin, tachykinins, oxytocin, endothelin-1, bombesin and bombesin-like peptides [7,10].

Aside from its ability to regulate the effect of biological factors through its enzymatic activity, several data suggest that both glycoproteins exert other functions which contribute to tumor etiopathogenesis. Thus, NEP can influence by itself some signal transduction pathways that regulate cell-growth, migration, and apoptosis [7], and DPP IV may work as a functional collagen receptor with roles in T-cell activation in thymic ontogeny [6] and also regulate tumor cell behavior through interaction with fibroblast activation protein-α[11].

DPP IV and NEP biological actions are being increasingly elucidated in the last years and their role in renal tumor genesis and development is an emerging issue with potential clinical implications

Some of our previous studies in this field have demonstrated that membrane-bound peptidases, including two glycoproteins (APA/gp160 and APN/gp150), could be involved in renal cancer etiogenesis. In particular, we have described a striking reduction in the activity of APA, APN and APB peptidases, which could be related to the histogenetic origin of the most frequent renal tumor subtypes [12,13].

In this manuscript, we present the metabolic and expression profiling of DPP IV and NEP glycoproteins in three main histological types of renal tumors (covering 80% of these neoplasms), namely CCRCC, ChRCC and RO. Additionally, this profiling is also presented in different CCRCC grades and stages, two key histopathological parameters for tumor prognosis [3].

## Methods

The authors declare that all the experiments carried out in this study comply with current Spanish and European Union laws and conform to the principles outlined in the Declaration of Helsinki.

### Renal tissue specimens and sample storage

We analyzed renal tissue in a series from 75 patients with CCRCC (60 male, 15 female; mean age: 63 years), 10 patients with ChRCC (4 male, 6 female; mean age: 65 years) and 8 patients with RO (6 male, 2 female; mean age: 68 years). Patient consent and Hospital Ethics Committee approval were obtained a priori. Fresh tissue samples were obtained from surgical specimens from renal tumor patients. Tumor and normal (surrounding uninvolved tissue) areas were obtained in all cases. For RT-PCR studies, tissue samples were immersed in RNAlater (Ambion, Huntingdon, UK) immediately after dissection and stored at -80°C until use. For activity studies, tissue samples were embedded in OCT, frozen in isopentane, and stored at -80°C until the enzyme assays were performed. In addition, selected tissue samples were formalin-fixed and paraffin-embedded for immunohistochemistry assay of DPP IV and NEP and for histopathological diagnosis. The 2004 WHO histological classification of adult renal cell tumors [3], the 2002 TNM Edition for tumor staging [14], and the Furhman's method for grading [15] were used for performing the histopathological diagnoses.

### Quantitation of DPP IV and NEP Catalytic Activity

#### Sample preparation

Explanted tissue samples were homogenized in 10 mM Tris-HCl buffer, pH 7.4, for 30 seconds at 800 rpm using a Heidolph PZR 50 Selecta homogenizer and ultracentrifuged in a Centrikon T-2070 Kontron Instruments apparatus at 100,000 g for 35 min. The resulting supernatants were used to measure soluble DPP IV activity, which is a truncated form of membrane-bound DPP IV/CD26 lacking some residues in the N-terminal aminoacids [16]. To avoid contamination with soluble enzymes, the resulting pellets were washed three times by suspension in 10 mM Tris-HCl buffer, pH 7.4. The pellets were then homogenized in 10 mM Tris-HCl buffer, pH 7.4, and centrifuged at low speed (1,500 g) for 1 min to purify the nucleus from the samples. The supernatants thus obtained were used to determine particulate enzyme activities and protein concentrations. All steps were carried out at 4°C.

### Enzyme assays

All reagents used in these assays were purchased by Sigma-Aldrich^®^, Spain division.

DPP IV activity was measured in triplicate by using H-Gly-Pro-β-naphthylamide as substrate, following the method of Liu and Hansen [17]. The NEP assay was carried out by incubating samples with a saturating concentration of N-Dansyl-D-Ala-Gly-pNOH2-Phe-Gly ([D]AG(pN)PG, a Dansyl derivative), following the method of Florentin et al. (1984) [18].

These assays are based on the fluorescence of products generated from the hydrolysis of the substrate by the enzyme. Reactions were initiated by adding 30-50 μL of sample to 1 mL of the appropriate incubation mixture (50 mM Tris-HCl buffer, pH 7.4, and 0.2 mM aminoacyl-β-naphthylamide or 0.125 mM [D]AG(pN)PG). After 30 min incubation at 37°C, 1 mL of 0.1 M sodium acetate buffer (pH 4.2) was added to the mixture to terminate the reaction. The released product was determined by measuring the fluorescent intensity (at 412 nm with excitation at 345 nm for β-naphthylamine, and at 562 nm with excitation at 342 nm for [D]AG) with a Shimadzu RF-540 Spectrofluorophotometer. Blanks were used to determine background fluorescence. The relative fluorescence was converted into picomoles of product using a standard curve constructed with increasing concentrations of β-naphthylamine or [D]AG.

#### Protein determination

Protein concentration was measured in triplicate by the method of Bradford, using BSA (1 mg/mL) as the calibrator. The results were recorded as units of peptidase (UP) per milligram of protein. One unit of peptidase activity is the amount of enzyme necessary to release one pmol of fluorogenic product per minute (pmol/min). Fluorogenic assays were linear with respect to hydrolysis time and protein content.

### Quantitation of DPP IV and NEP mRNA expression

#### RNA isolation

Quantitative RT-PCR for detecting DPP IV and NEP mRNA (*DPP4 *and *MME *genes respectively) was performed to assess the transcription levels of these peptidases. To avoid any RNA degradation only samples that were immediately immersed in RNAlater after dissection were used in these experiments. Hence, total RNA of tumor and non-tumor tissue samples from 26 CCRCC (21 male, 5 female; mean age: 63 years), 6 ChRCC (2 male, 4 female; mean age: 63 years) and 4 RO (4 male, 0 female; mean age: 63 years) patients was isolated following a standard protocol as previously described [19]. Briefly, after homogenization of the samples, total RNA was isolated with TRIzol Reagent (Invitrogen Life Technologies, Karlsruhe, Germany) according to the manufacturers manual, using approximately 50 mg frozen tissue per milliliter TRIzol.

#### Real-time quantitative RT-PCR Analysis

First-strand cDNA was synthesized from 25 μg of total RNA of each human sample using Moloney murine leukemia virus reverse transcriptase and random hexamers according to the manufacturer's instructions (First-strand cDNA Synthesis Kit, Amersham Biosciences, Essex, UK). The resulting cDNA samples were amplified by PCR with specific oligonucleotide primer pairs designed with the analysis software Primer 3 [20]. Based on previous experiments on human renal cell carcinoma [19,21] and other human tissues [22,23] we chose TATA box binding protein (*TBP*), peptidylprolyl isomerase A (*PPIA*), β-actin (*ACTB*) and succinate dehydrogenase complex, subunit A (*SDHA*) as endogenous reference genes. The sequence of the primers used to amplify *DPP4*, *MME *and the four housekeeping genes are shown in Table 1. All primers were synthesized and purified by Sigma-Genosys (Cambridge, UK).

**Table 1:** Sequence of forward (F) and reverse primers (R) of indicated target genes and the size expected for each PCR-amplified product.

*Peptidase*	*Gene symbol*	*Fordward primer*	*Reverse primer*	*Amplicon size (bp)*
DPPIV/gp110	*DPP4*	5'-AGTGGCGTGTTCAAGTGTGG-3'	5'-CAAGGTTGTCTTCTGGAGTTGG-3'	112
NEP/CALLA glycoprotein	*MME*	5'-CCGAGAAAAGGTGGACAAAGA-3'	5'-GGACTGCTGGGCACTAAAGAA-3'	133

*Housekeeping Gene name*				

β-actin	*ACTE*	5'-TCCCTGGAGAAGAGCTACGA-3'	5'-ATCTGCTGGAAGGTGGACAG-3'	362
Succinate dehydrogenase complex, subunit A	*SDHA*	5'-TCTGCCCACACCAGCACT-3'	5'-CCTCTCCACGACATCCTTCC-3'	142
TATA box binding protein	*TBP*	5'-GGATAAGAGAGCCACGAACCAC-3'	5'-TTAGCTGGAAAACCCAACTTCTG-3'	139
Peptidylpropyl isomerise A	*PPIA*	5'-GGTCCCAAAGACAGCAGAAAA-3'	5'-TCACCACCCTGACACATAAACC-3'	114

Primers for the assayed housekeeping genes are also shown.

The expression of the housekeeping genes, *DPP4 *and *MME *was quantified in all cDNAs by real-time PCR using the iCycler iQ real-time detection apparatus (BioRad Laboratories, Hercules, CA, USA). Dilutions of the cDNA template were prepared from each tissue and amplified in triplicate using SensiMix Plus SYBR + FLUORESCEIN (Quantace Ltd., London, UK). Three negative controls (with no template, no reverse transcriptase and no RNA in the reverse transcriptase reaction) were also included in each plate to detect any possible contamination. After a hot start (10 min at 94°C), the parameters used for PCR amplification were: 10 s at 94°C, 20 s at 60°C and 30 s at 72°C, for 50 cycles.

Real-time PCR data were expressed as the fold change of the target gene expression relative to the geometric mean (g.m.) mRNA expression of the housekeeping genes in each sample, as described by Vandesompele et al. [24]. The fold change in gene expression was calculated by the formula: 2^-ΔC^_T_, where C_T _is the threshold cycle, calculated by the iCycler software, ΔC_T _= (C_Ttarget gene _- C_Tg.m. reference genes_) and ΔΔC_T _= (ΔC_Ttest sample _- ΔC_Tcontrol sample_).

For each type of renal tumor, paired malignant (tumor) and uninvolved surrounding samples (normal) from the same patient were always measured in the same analytical run to exclude between-run variations. PCR data obtained in one of the normal kidney samples were arbitrarily chosen as control, and this sample was included in all PCR experiments to correct for possible interassay variations. As was for the enzymatic activities, an additional assay-set was performed to compare expression levels of *MME *and *DPP4 *between different grades and stages in CCRCC samples.

As indicated in previous works, specifically in the field of RCCs, expressions measured in these systems may not always be normally distributed. Thus, we performed a D'Agostino & Pearson omnibus normality test. Since expression data for the three tumor types fitted to a Gaussian distribution, the unpaired t test was applied for all significance calculations.

### Semiquantitative evaluation of DPP IV and NEP expression in renal tumors

#### Immunohistochemistry

Conventional tissue samples from patients with different tumor-phenotypes (CCRCC; ChRCC; RO) and their normal tissues were subjected to immunohistochemistry using a rabbit polyclonal antibody specific for DPP IV/CD26 (Abcam plc, Cambridge, UK) at 1:250 working dilution, and a rabbit monoclonal antibody specific for NEP/CD10 (Novocastra, Newcastle, UK) at 1:30 working dilution.

Briefly, endogenous peroxidase activity was blocked by incubating the slides in 3% hydrogen peroxide in absolute methanol for 10 minutes. Antigen retrieval was carried out in citrate buffer (10 mM, pH = 6) for 15 minutes at 100°C in a microwave oven. The primary antibody was applied for 1 hour at room temperature. A subsequent reaction was performed with secondary antobodies and biotin-free HRP enzyme labeled polymer of the EnVision-plus detection system (Dako, Carpinteria, CA). Nonspecific IgG was used as a negative control. A positive reaction was visualized with diaminobenzydine solution followed by counterstaining with hematoxylin.

#### Clinical evaluation

All the sections were evaluated microscopically by using conventional techniques in the Laboratory of Anatomic Pathology. A section was considered negative or positive according to the absence or presence of staining. Two independent observers blinded from the clinical data analyzed separately the immunostaining reactions for both peptidases. Positive immunoreactivity was assessed by the relative intensity of staining in the sample. Thus the immunostaining results were divided into 4 categories: negative (-; lack of specific staining); mild staining (+); moderate staining (++); intense staining (+++). Respectively, each positive staining was subdivided into 2 categories: diffuse staining; focal staining.

#### Statistical analyses

Data were analyzed statistically using SPSS^©^. Unpaired Student's t test was performed to detect differences between uninvolved tissues and tumors, as well as among low and high grades and stages. Statistically significant differences were considered at p < 0.05.

## Results

### DPP IV and NEP activity profile in renal tumors

**Figure 1:**
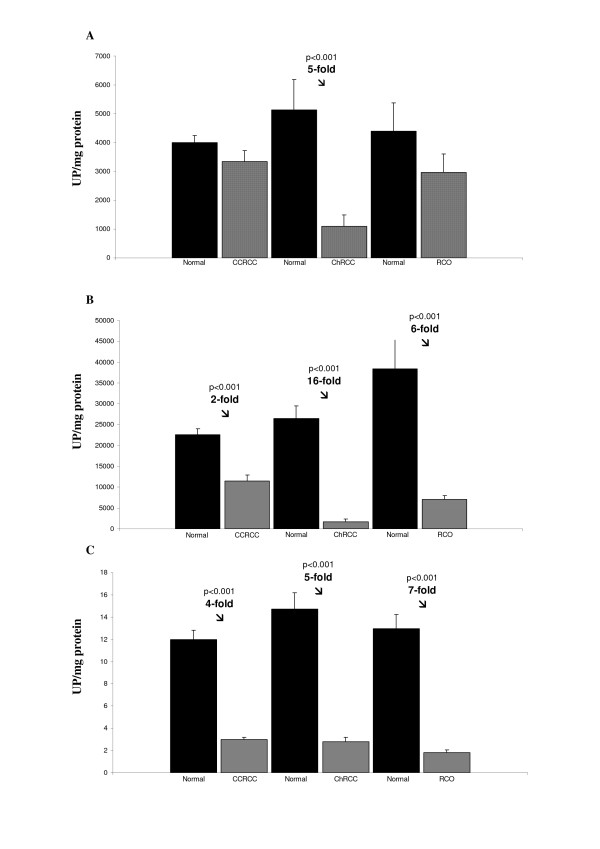
**Soluble (A) and membrane-bound DPP IV (B), and NEP (C) peptidase activity profiles in clear cell renal cell carcinoma (CCRCC), chromophobe renal cell carcinoma (ChRCC) and renal oncocytoma (RO)**. Columns compare tumor with non-tumor surrounding tissue (normal). Values represent mean ± SE of units of peptidase (UP) per milligram of protein.

**Figure 2:**
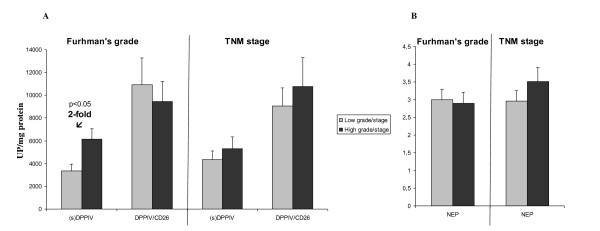
**Soluble (s) and membrane-bound DPP IV (A), and NEP (B) peptidase activity profiles in different grades and stages of clear cell renal cell carcinoma (CCRCC)**. Columns compare high grade (G3-G4) and stage (T3-T4) tumors with low grade (G1-G2) and stage (T1-T2) tumors. Values represent mean ± SE of units of peptidase (UP) per milligram of protein.

Data obtained in the activity assays of both glycoproteins across the different tumor types and in stratified CCRCC are given in Figures 1 and 2.

Figure 1 shows DPP IV and NEP activities measured in tumor and non-tumor tissue (normal) of CCRCC (n = 75), ChRCC (n = 10) and RO (n = 8) patients. Activity is recorded as pmol of product/min/mg protein (UP/mg protein) and presented as mean ± SE.

As shown in Figure 1A, when compared with non-tumor tissues, soluble DPP IV activity decreased significantly (fivefold) only in chromophobe carcinomas. Values for the soluble DPP IV activities in CCRCC and in RO did not vary significantly.

The membrane-bound DPP IV activity in renal tumors (Figure 1B) decreased significantly in all tumor types we analyzed (CCRCC, ChRCC and RO) when compared with the normal tissue samples. Loss of activity was slight in CCRCC (twofold), whereas it drastically decreased in the ChRCC (sixteenfold) and, although in a lesser intensity, in the RO (sixfold).

With respect the NEP activity, it is shown in Figure 1C. As in the case of the aforementioned membrane-bound glycoprotein, NEP activity decreased significantly in CCRCC (fourfold), ChRCC (fivefold) and RO (sevenfold) when compared with the normal tissue samples. We did not detect any NEP activity in the soluble fraction (data not shown).

Figure 2 represents DPP IV and NEP activity in the different stages and grades of CCRCC group (Low grade: G1-G2, n = 38 vs High grade: G3-G4., n = 37; Low stage: T1-T2, n = 48 vs High stage: T3-T4, n = 27). Activity is recorded as pmol of product/min/mg protein (UP/mg protein) and presented as mean ± SE.

The study of DPP IV yielded significant results (Figure 2A). After stratification by grade, soluble (s)DPP IV activity was twofold significantly decreased in CCRCCs with a low Furhman's grade in comparison to those clear cell carcinomas in a higher grade. Statistical analyses of grading for cell surface DPP IV (DPP IV/CD26) and staging for both soluble and membrane-bound DPP IV activities were not significant.

In Figure 2B, NEP activities in graded and staged CCRCCs are shown. No significant changes were found after stratification of this activity.

### Quantitative DPP IV and NEP expression profile in renal tumors

**Figure 3:**
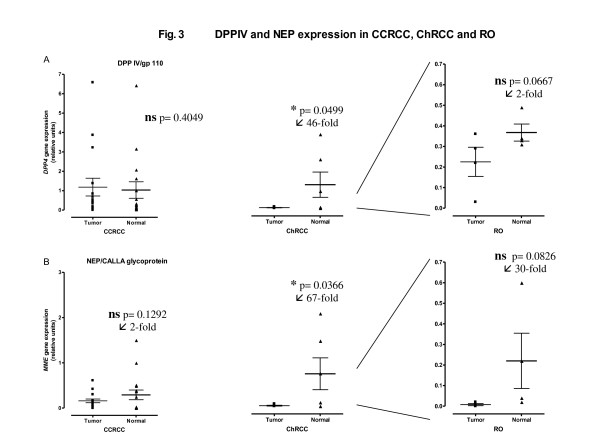
**mRNA levels of DPP IV (A) and NEP (B) measured in tumour and nontumour tissue (normal) for CCRCC (n = 16), ChRCC (n = 6) and RO (n = 4) patients**. RT-PCR data for each analyzed sample are recorded as relative units. The mean ± SE, p values and "normal/tumour" ratio of expression levels are also represented. *Statistically significant. **ns **no significant.

**Figure 4:**
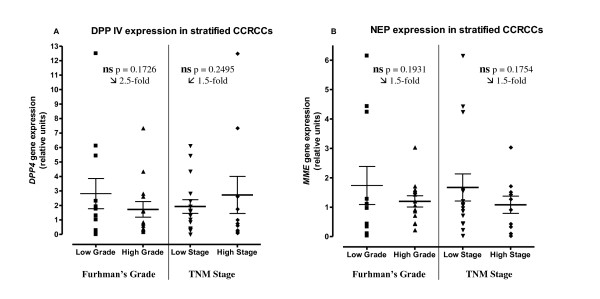
**mRNA levels of DPP IV (A) and NEP (B) measured in different grades and stages of clear cell renal cell carcinoma (CCRCC)**. RT-PCR data for each analyzed sample are recorded as relative units. The mean ± SE, p values and "normal/tumour" ratio of expression levels are also represented. *Statistically significant. **ns **no significant.

Data obtained in the qRT-PCR assays of both glycoproteins across the different tumor types and in stratified CCRCC are given in Figures 3 and 4.

Figure 3 shows the DPP IV and NEP mRNA levels measured in tumour and nontumour tissue (normal) for CCRCC (n = 26), ChRCC (n = 6) and RO (n = 4) patients. RT-PCR data for each analyzed sample are recorded as relative units, as calculated by the ΔΔC_T _method. The mean ± SE, p values and "normal/tumour" ratio of expression levels are also represented.

Significant decreases of expression levels were only observed in the ChRCC when compared with the normal tissue. Thus, DPP IV relative expression (Figure 3A) decreased fortysixfold in chromophobe RCCs, whereas NEP mRNA levels (Figure 3B) decreased sixtysevenfold in the same tumor-type.

Changes were not significant for DPP IV (Figure 3A) nor NEP expression (Figure 3B) in CCRCC and RO tumors when compared with their corresponding normal tissues. However, such as in activity assays, a decreasing trend of mRNA levels was also observed for DPP IV in RO and for NEP in CCRCC and RO. Thus, as shown in Figure 3A, DPP IV expression did not change in CCRCC, and it decreased twofold in RO. NEP expression levels (Figure 3B) decreased twofold in CCRCC and thirtyfold in RO.

Figure 4 shows the DPP IV and NEP mRNA levels measured in the different grades and stages of CCRCC group (Low grade: G1-G2, n = 12 vs High grade: G3-G4, n = 14; Low stage: T1-T2, n = 16 vs High stage: T3-T4, n = 10). RT-PCR data for each analyzed sample are recorded as relative units, as calculated by the ΔΔC_T _method. The mean ± SE, p values and "normal/tumour" ratio of expression levels are also represented.

We did not find significant variations of expression in CCRCC related to its stratification in grades and stages, either in DPP IV (Figure 4A) nor in NEP mRNA levels (Figure 4B).

### Membrane-bound protein expression (Semiquantitative Immunostaining)

**Figure 5:**
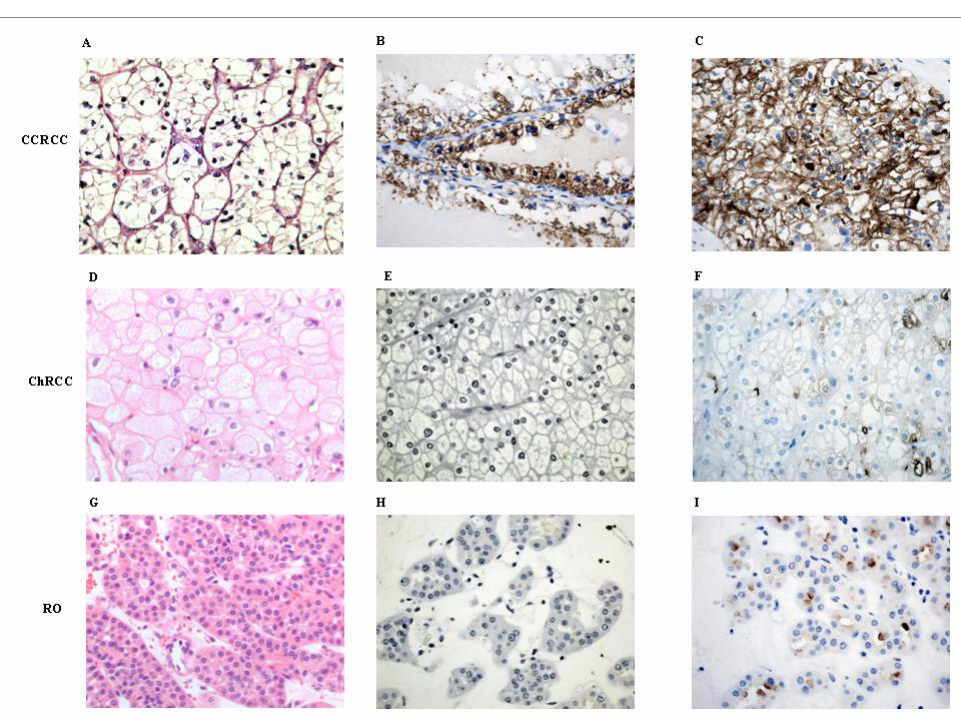
**Immunohistochemistry of DPP IV and NEP in renal tumors**. Hematoxylin and eosin staining of CCRCC (A), ChRCC (D) and RO (G). DPP IV/CD26 staining of CCRCC (B), ChRCC (E) and RO (H). NEP staining of CCRCC (C), ChRCC (F) and RO (I). Tissues magnification at 400×.

**Figure 6:**
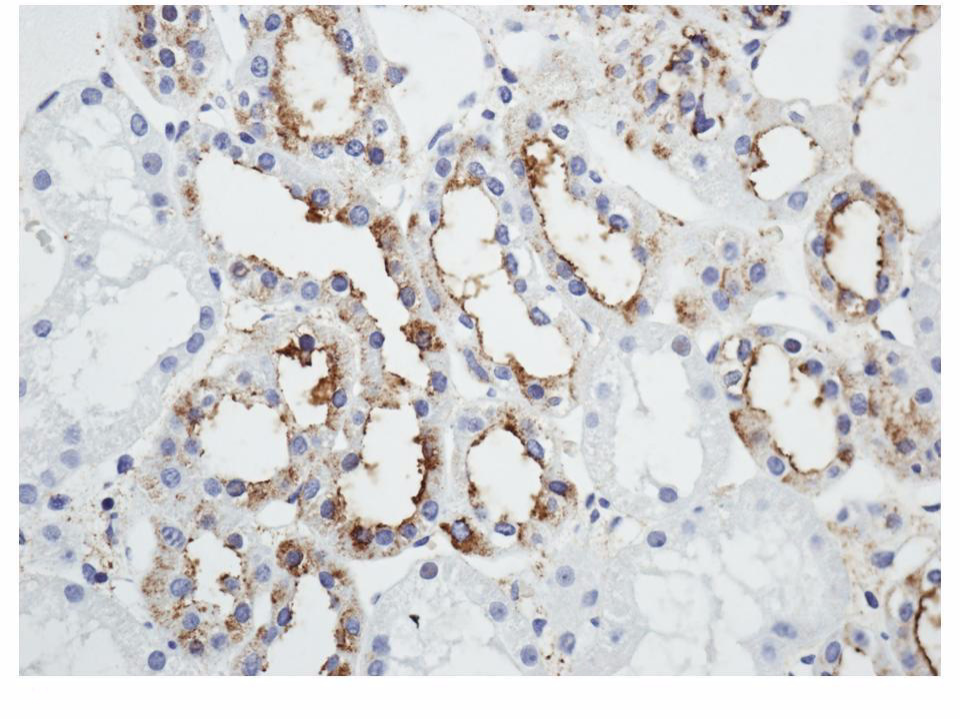
**Immunohistochemistry of DPP IV and NEP in normal renal tissue**. DPP IV immunostainings are selectively located in proximal tubules. Tissues magnification at 400×

**Figure 7:**
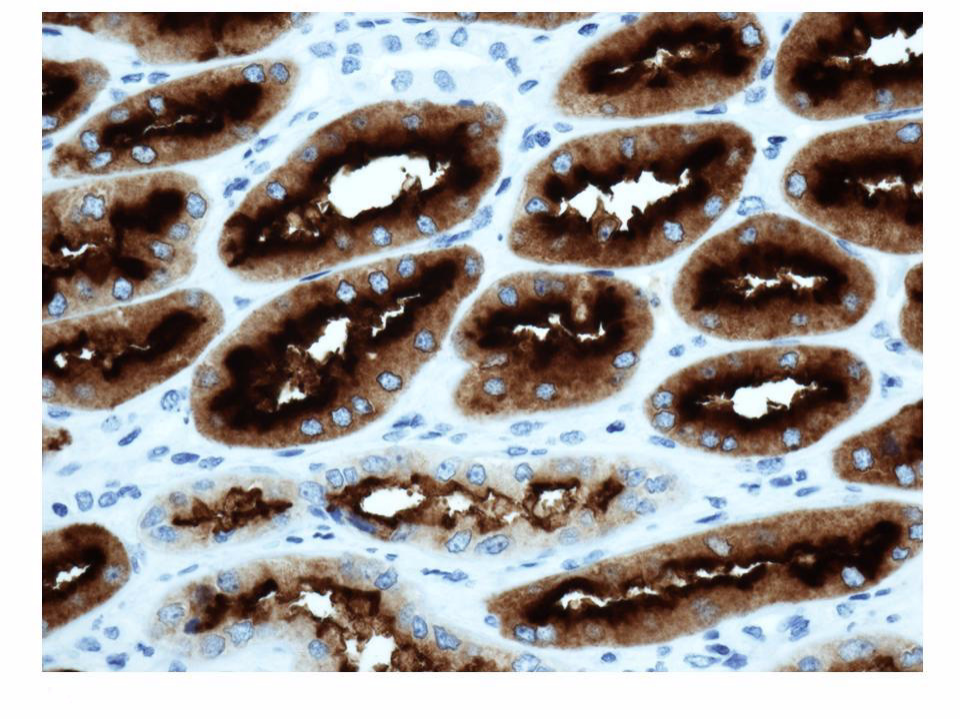
**Immunohistochemistry of DPP IV and NEP in normal renal tissue**. NEP immunostainings are selectively located in proximal tubules. Tissues magnification at 400×

DPPIV and NEP immunostaining distribution in renal tumours and normal kidney is presented in Table 2 and in Figures 5, 6 and 7. Semiquantitative features recorded in this table were the relative intensity of specific staining for both DPP IV and NEP glycoproteins (negative, mild, moderate, intense), and the distribution of that staining (diffuse, focal), in tumor types.

**Table 2:** Semiquantitative evaluation of renal tumors. (-) Negative immunostaining; (+) Mild immunostaining; (++) Moderate immunostaining; (+++) Intense immunostaining.

	DPP IV/CD26 immunostaining	NEP/CD10 immunostaining
**Tumor tissue**	**Staining-intensity**	**Staining-intensity**

**CCRCC**	++ diffuse	+++ diffuse
**ChRCC**	-	+ focal
**RO**	-	+ focal

Normal tissue	+	+++

DPP IV specific staining was moderate and diffuse in the CCRCC (Figure 5B), however there was not immunoreaction to this cell-surface protein in the ChRCC (Figure 5E) nor in the RO (Figure 5H).

In contrast, immunostaining for NEP was positive in all the tumor-types we analyzed. Thus, NEP staining was intense and diffuse in the CCRCC (Figure. 5C), whereas it was mild and focal in both ChRCC (Figure 5F) and RO (Figure 5I).

A positive immunostaining with DPPIV/CD26 (Figure 6) and NEP/CD10 (Figure 7) was found in the proximal tubules of normal kidney. Conversely, distal tubules were negative.

## Discussion

In this manuscript we assessed DPP IV and NEP catalytic activitiy, membrane-bound expression, and mRNA levels in a subset of renal tumors and found that both glycoproteins were selectively altered in neoplastic tissue. Enzyme activities were significantly decreased in the tumor tissue of all histological types, a trend which was especially sharp in ChRCC and RO. With respect to protein expression, DPP IV and NEP were down-regulated in ChRCC and RO, whereas CCRCC showed a moderate to strong immunostaining. This pattern was similar to that observed at mRNA levels. Thus, the relative expression of *DPP4 *(DPP IV transcriptome) and *MME *(NEP transcriptome) genes were found to be strongly decreased in ChRCC samples (DPP IV: ↓ fortysixfold, NEP: ↓ sixtysevenfold; tumor vs normal) and, although no statistically significant, slightly to strongly down-regulated in RO (DPP IV: ↓ twofold, NEP: ↓ thirtyfold; tumor vs normal). In contrast, mRNA levels for both glycoproteins did not significantly vary in CCRCC when compared with its normal tissue.

A main result in this work is that both glycoproteins showed a distinct pattern when compared tumors with the normal sorrounding tissues. DPP IV and NEP activities were markedly decreased in all tumor subtypes, and protein and mRNAs were strongly down-regulated in ChRCC and RO. These results agree with previous studies indicating that modifications in the activity and expression profiles of DPP IV and NEP are key events in malignant tumors, pointing to an involvement of these proteins in tumor cell growth, local invasion and metastasis [7,25-27]; and, in addition, this study extend that role of both peptidases to the renal tumors. Moreover, the present manuscript shows that the modifications affecting DPP IV and NEP profiles along the different phenotypes of renal cancer are similar to those we observed in our previous studies on other membrane-bound peptidases, such as IRAP, APN and APA [12,13,28], and thus reinforces the idea that loss of several physiologically significant glycopeptidases may be a critical step in the etiogenesis of renal tumors. To support this fact, it has been described that membrane-bound ectopeptidases can affect, in solid tumors, cellular events classically shown to be influenced by matrix metalloproteinases (MMPs) and other secreted proteases [27].

This study also demonstrates a different DPP IV and NEP protein and mRNA expression depending on tumor type. Immunostaining in normal renal tissue revealed that both enzymes were exclusively located in the proximal nephron, the proposed site of origin of CCRCC [3], which showed moderate to intense positivity. Conversely, protein expression and mRNA levels of tumors supposedly derived from the distal nephron (ChRCC and RO) were markedly down-regulated. This work agrees with previous studies [29-34] and further supports the theory of the different origin of tumors along the nephron proposed in the 2004 WHO classification [3].

The exact role that DPP IV plays in different cancers remains unclear, partially due to its variable expression along the different tumor-types. Thus, both up- and down-regulation of this protein have been described depending on the studied tumor and organ [6,26]. With respect to the relationship between DPP IV expression and cancer, the unique example of a clear causal effect is seen in human melanocytes where the loss of DPP IV is invariably associated with malignant transformation [35]. We also have observed a down-regulation in the activity and expression of this glycoprotein in renal tumors, and in this sense our results agree with those described in the human melanoma [36].

DPP IV is a glycoprotein which presents demonstrated pleiotropic effects, and it is likely that this multifunction accounts for its varied roles in different cancers. Thus, there may be two main mechanisms by which DPP IV affects cellular function: on one hand its catalytic activity on bioactive peptides and, on the other hand, its direct interaction with certain molecules located outside the cells [9,26,36]. This feature makes difficult to ascertain which is the exact way DPP IV plays in cancer, and further investigations are required to elucidate the concrete molecular mechanisms of this glycopeptidase in renal tumor biology.

DPP IV has been commonly described as a membrane-bound peptidase, but the expression and activity of soluble isoforms have been reported in body fluids and in cytosolic fractions [6,37,38]. Previous studies have reported altered soluble DPP IV activities in several neoplasms, suggesting the potential value of this enzyme as a prognostic variable of cancer patients [6,39]. In this sense, we have found that soluble DPP IV activity significantly decreased (twofold) in low grade CCRCCs (G1-G2) when compared with their more aggressive counterparts (high grade: G3-G4). In addition, although no statistically significant, we also observed a decreasing trend between activities of different CCRCC stages in both soluble (22%↓; low vs high stage) and membrane-bound DPP IV (19%↓; low vs high stage). A similar phenomenon has been observed previously with other peptidases and kallikreins in renal carcinomas [13,36,40], suggesting that these proteases may predict a poor disease outcome in RCC.

Several authors have referred to the potentiality of NEP/CD10 as diagnostic marker in the RCCs [29,30]. Thus, NEP (CD10) is a useful immunohistochemical marker in the identification of proximal nephron-derived carcinomas such as CCRCC [31], and usually shows negative immunostaining in distal nephron-derived tumors like ChRCC [32]. However, although the immunohistochemical expression patterns of NEP along the renal cancer have been broadly documented, works on activity and mRNA profiles of this glycoprotein in renal tumors are lacking. In this sense, our results add new findings to understand the role of NEP in RCCs.

Loss or decrease in NEP expression has been reported in several cancer types, such as invasive bladder carcinoma, poorly differentiated gastric adenocarcinoma, small cell and non-small cell lung carcinomas, endometrial adenocarcinoma and prostate adenocarcinoma [41,42]. Our data on NEP activity, immunostaining and mRNA agree with general findings about these enzymes in the human neoplasia, extending this knowledge to renal tumors as previously reported [43].

NEP has also been demonstrated to be a multifunctional glycoprotein. It is accepted that the interactions of this membrane-bound peptidase with other transmembrane proteins and/or the extracellular matrix (ECM) may have similar or even more relevance in regulating cells than cleaving bioactive peptides [7]. NEP is considered to be a tumor suppressor protein which, in addition, interacts with other tumor suppressors such as PTEN [7,44], demonstrating an anti-angiogenic effect [42]. Since, as we observed, a strong decrease in NEP activity and expression appears to be a common feature in the renal tumor etiogenesis, our data may support a potential anti-tumor function of NEP in renal cancer.

## Conclusion

This work demonstrates a strong downregulation of DPP IV and NEP in the renal tumors, different protein and mRNA expression profiles, which depend on the tumor type, and a positive correlation between soluble DPP IV activity and aggressiveness in CCRCCs. These results support the idea of a pivotal role for DPP IV and NEP in the malignant transformation of renal neoplasms and stress the importance of both glycoproteins as potential diagnostic tools. Further studies comparing enzymatic activities and expression profiles with patient survival will help us to determine the approppriate use of DPP IV and NEP also as prognostic tools.

## Nonstandard Abbreviations

CALLA: (common acute lymphoblastic leukemia antigen); CCRCC: (clear-cell renal cell carcinoma); ChRCC: (chromophobe renal cell carcinoma); DPPIV: (dipeptidyl peptidase IV); NEP: (neprilysine, neutral endopeptidase); RO: (renal oncocytoma); UP: (Units of Peptidase, pmol of product/min).

## Competing interests

The authors declare that they have no competing interests.

## Authors' contributions

AV contributed to the study design, supervised assays, interpreted the results and drafted the manuscript. LB and IP carried out fluorimetric and qRT-PCR assays, and contributed to bio-statistical analysis. JG and JI developed the protocol of enzymatic assays and contributed to the study design. JIL designed and carried out immunohistochemical study, interpreted results and contributed in the paper writting. MLC and FMP developed the protocol of the qRT-PCR study and interpreted the results. GL contributed to the study design, supervised assays, interpreted the results and contributed in the paper writing. All authors read each draft and approved the final manuscript.

## Pre-publication history

The pre-publication history for this paper can be accessed here:

http://www.biomedcentral.com/1471-2407/10/193/prepub
